# Characterization of community-associated *Staphylococcus aureus* from skin and soft-tissue infections: a multicenter study in China

**DOI:** 10.1038/emi.2016.128

**Published:** 2016-12-21

**Authors:** Ying Liu, Zhe Xu, Zhou Yang, Juan Sun, Lin Ma

**Affiliations:** 1 Department of Dermatology, Beijing Children's Hospital, Capital Medical University, Beijing 100045, China

**Keywords:** methicillin-resistant *Staphylococcus aureus*, China, molecular epidemiology, skin and soft-tissue infection, *Staphylococcus aureus*

## Abstract

We evaluated the epidemiological and molecular features of community-associated methicillin-resistant *Staphylococcus aureus* (CA-MRSA) and methicillin-sensitive *S. aureus* (MSSA) from children and adult patients with skin and soft-tissue infections (SSTIs) in China. Prospective community-acquired *S. aureus* SSTI surveillance was conducted in 23 hospitals over a 24-month period. Susceptibility to 16 antimicrobials was evaluated using the agar dilution method. StatApriori was used to determine statistically significant association trends. The genotypic characteristics of CA-MRSA isolates were tested by staphylococcal cassette chromosome *mec* (SCC*mec*) typing, staphylococcal protein A (*spa*) typing, and multilocus sequence typing. The presence of Panton–Valentine leukocidin (*pvl*) genes was determined. Overall, 71.6% (1946/2716) of cases were community-associated *S. aureus*. CA-MRSA accounted for 2.6% (51). Out of 1895 methicillin-sensitive *S. aureus* strains, 97.3% were resistant to erythromycin, 96.6% to penicillin and 89.1% to clindamycin. No *S. aureus* strains were resistant to vancomycin. Thirteen sequence types (STs) and 17 *spa* types were detected among the CA-MRSA strains. The most prevalent sequence type was ST121 (19/51, 37.3%), followed by ST59 (13/51, 25.5%). In addition, t437 was predominant, accounting for 43.1% (22/51). Only five (9.8%) of the CA-MRSA strains harbored *pvl* genes. There were no significant differences in antibiotic sensitivity profiles between ST121 and non-ST121 MRSA isolates. However, ST121 strains tended to be more resistant to cefazolin, whereas non-ST121 strains were more resistant to chloramphenicol. In conclusion, CA-MRSA infections are rare among Chinese SSTI patients. MRSA strains in China have diverse genetic backgrounds, with ST121 being the predominant clone. Fusidic acid and mupirocin remain effective for topical treatment.

## INTRODUCTION

*Staphylococcus aureus (S. aureus)* is a significant human pathogen worldwide, particularly in healthcare settings. It has long been recognized as an important causative agent of skin and soft-tissue infections (SSTIs), which range from folliculitis to life-threatening conditions such as necrotizing fasciitis. The increasing prevalence of methicillin-resistant *S. aureus* (MRSA) and its ability to resist multiple drugs has posed a serious challenge.^1^ Hospital-associated MRSA has been extensively investigated, with a high prevalence of MRSA (52.3%–76.9%) in mainland China.^2^ However, considering China's vast territory, the data on SSTIs caused by community-associated methicillin-resistant *S. aureus* (CA-MRSA) are limited. Despite the importance of MRSA, methicillin-sensitive *S. aureus* (MSSA) is among the most common causative agents of SSTIs.^3^ The epidemiological surveillance of MRSA and MSSA is critical for the development and implementation of infection control programs.

This study focused on an expanded sample of SSTI cases to obtain accurate molecular characteristics of CA-MRSA in mainland China and to produce a more comprehensive national description of the molecular epidemiology and resistance profiles of CA-MRSA in children and adults in China. Although previous studies reported on *S. aureus* biogeography and virulence,^4^ to our knowledge, this is the first study to compare contemporaneous CA-MRSA of SSTIs from adults and children and the first such study carried out in mainland China.

## MATERIALS AND METHODS

### Patient enrollment

This was a laboratory-based multicenter study involving the voluntary participation of 23 hospitals (including 13 children's hospitals and ten adult hospitals) in six geographical regions (Northeast, North China, Northwest, East China, South Central China and Southwest) located throughout China. The inclusion criteria for these institutions were: (i) at least one children's hospital and one adult hospital located in five regions, but only one children's hospital in the Northeast area; and (ii) certified as a rank A tertiary hospital by the Ministry of Health of China. Clinical data were collected from outpatients with SSTIs in the Department of Dermatology of each hospital during a 24-month period from October 2009 to September 2011.

### Clinical data collection

Eligible patients were those with at least one sample from any site that was culture positive for *S. aureus* who met the following criteria for community-associated *S. aureus*: (i) a culture sample was obtained during an outpatient visit or within 48 h of hospitalization; (ii) the patient had not been admitted to a hospital, nursing home or any other long-term care facility within the past year; and (iii) the patient had no history within the past year of known risk factors for MRSA, including current intravenous drug use, surgery, dialysis, an indwelling catheter or a percutaneous medical device. A case report form was completed for each patient who included demographic information, clinical symptoms, laboratory findings, type of infection diagnosed, all antibiotic use and clinical outcome.

### Bacterial strains

Specimens were collected from infection sites of every patient enrolled and cultured on blood agar. Preliminary identification was performed based on bacterial morphology, Gram staining, hemolysis, and catalase tests at the central laboratory. Then, Slidex Staph Plus (bioMérieux, Marcy I'Etoile, France) latex agglutination was performed for the rapid detection of *S. aureus*. To avoid overrepresentation, we included only the first isolate from each patient. MRSA isolates were initially identified using the oxacillin minimum inhibitory concentration method and confirmed for the presence of the *mecA* gene by PCR as previously described.^5^

### Antimicrobial susceptibility testing

The minimum inhibitory concentrations of penicillin, oxacillin, cefazolin, cefuroxime, ceftriaxone, erythromycin, clindamycin, ciprofloxacin, chloramphenicol, gentamicin, rifampicin, tetracycline, trimethoprim-sulfamethoxazole, vancomycin, fusidic acid and mupirocin were determined by agar dilution method in accordance with the 2010 guidelines of the Clinical and Laboratory Standards Institutes.^6^ All antibiotics were from Sigma Chemical Co., St Louis, MO, USA except mupirocin from GlaxoSmithKline, Brentford, London. Fusidic acid minimum inhibitory concentrations were determined according to the European Committee on Susceptibility Testing guidelines (EUCAST, version 1.3, 2010, Basel, Switzerland). The *S. aureus* strain ATCC 29213 was used as a control.

### Molecular typing methods

Staphylococcal cassette chromosome *mec* (SCC*mec*) typing, staphylococcal protein A (*spa*) typing, and multilocus sequence typing were performed using PCR as previously described for all CA-MRSA isolates.^5, 7, 8^ The presence of Panton–Valentine leukocidin (*pvl)* genes, such as lukS-PV and lukF-PV, was also determined by PCR as previously described.^9^

### Statistical analysis

A *χ*^2^-test or Fisher's exact test was used to analyze quantitative variables. Statistical analyses were performed using SPSS, version 13.0 software (SPSS, Chicago, IL, USA). A *P*-value of ≤0.05 was considered statistically significant. All susceptibility data and molecular test results were analyzed using WHONET software, version 5.6. StatApriori (WHO, Geneva, Switzerland) was used for searching statistically significant association rules.

## RESULTS

### Patient demographics

A total of 2716 patients were identified during the study period. Out of these patients, 71.6% (1946) fulfilled the criteria for inclusion in the study, 2.6% (51) met the definition of CA-MRSA infection and the rest were classified as having MSSA infection. The incidence of *S. aureus* from children's hospitals (82.6%, 1705/2046) was much higher than from adult hospitals (36.96%, 241/652). Common lesions in children included impetigo (81.9%, 1397/1705), staphylococcal scald skin syndrome (4.8%, 82/1705), and secondary infection of eczema (4.3%, 74/1705), whereas in adults, common lesions included secondary infection of eczema (24%, 58/241), impetigo (20%, 48/241) and folliculitis (11%, 27/241). Demographic and clinical features of all patients are shown in Table 1.

### Comparison of resistance profiles of *S. aureus* isolates

Overall, CA-MRSA accounted for 2.6% (51) of 1946 *S. aureus* strains. Of 1895 MSSA strains isolated, 97.3% strains were resistant to erythromycin, followed by 96.6% to penicillin, 89.1% to clindamycin, 42.0% to tetracycline and 14.4% to chloramphenicol. None of the *S. aureus* strains were resistant to vancomycin. The antimicrobial susceptibilities of MRSA isolates were compared with MSSA isolates (Table 2). CA-MRSA isolates demonstrated a higher rate of resistance to chloramphenicol, ciprofloxacin, ceftriaxone and cefixime (31.4% vs. 14.4%, 13.7% vs. 6.3%, 23.5% vs. 0.9% and 19.6% vs. 2.5%, respectively). The susceptibility profiles of MRSA to other antimicrobial agents were similar to those of MSSA. All CA-MRSA and MSSA isolates tested were sensitive to vancomycin. Significant differences in antimicrobial susceptibility profiles of isolates from children and adults were also observed (*P*<0.05) (Table 2). *S. aureus* isolates obtained from children were more likely to be susceptible to chloramphenicol, gentamicin, ciprofloxacin, trimethoprim-sulfamethoxazole, fusidic acid, mupirocin and cephalosporin than those from adults.

### Molecular characteristics of CA-MRSA isolates

High genetic diversity was observed among the 51 CA-MRSA isolates: 13 STs and 17 *spa* types harbored two SCC*mec* types (Table 3). Surprisingly, the most prevalent ST was ST121 (19/51, 37.3%), followed by ST59 (13/51, 25.5%). Among them, t437 was predominant, which accounted for 43.1% (22/51) of all CA-MRSA isolates, followed by t2086, t1425 and t127. Only SCC*mec* type IV was found in North China. Interestingly, strains ST121, ST59 and t437 were uncommon in East China. Some spatial variations were observed in the distribution of *spa* types (e.g., t437 occurred in 66.7% and 55.6% isolates from Children's Hospital of Chongqing Medical University and Beijing Children's Hospital, respectively). However, such differences were not significant. No other clinical or spatial associations were observed in the distribution of *spa* types or STs with regard to infection type, sex, region or hospital of origin.

Of the isolates tested, only five (9.8%) of the CA-MRSA isolates harbored *pvl* genes. Four of the strains were isolated from children. Three patients suffered from abscesses, one from a furuncle and one from cellulitis. Four of the strains were t437. The clinical and molecular characteristics of the five CA-MRSA isolates are shown in Table 3.

### Characteristics of ST121 CA-MRSA strains

**Figure 1:**
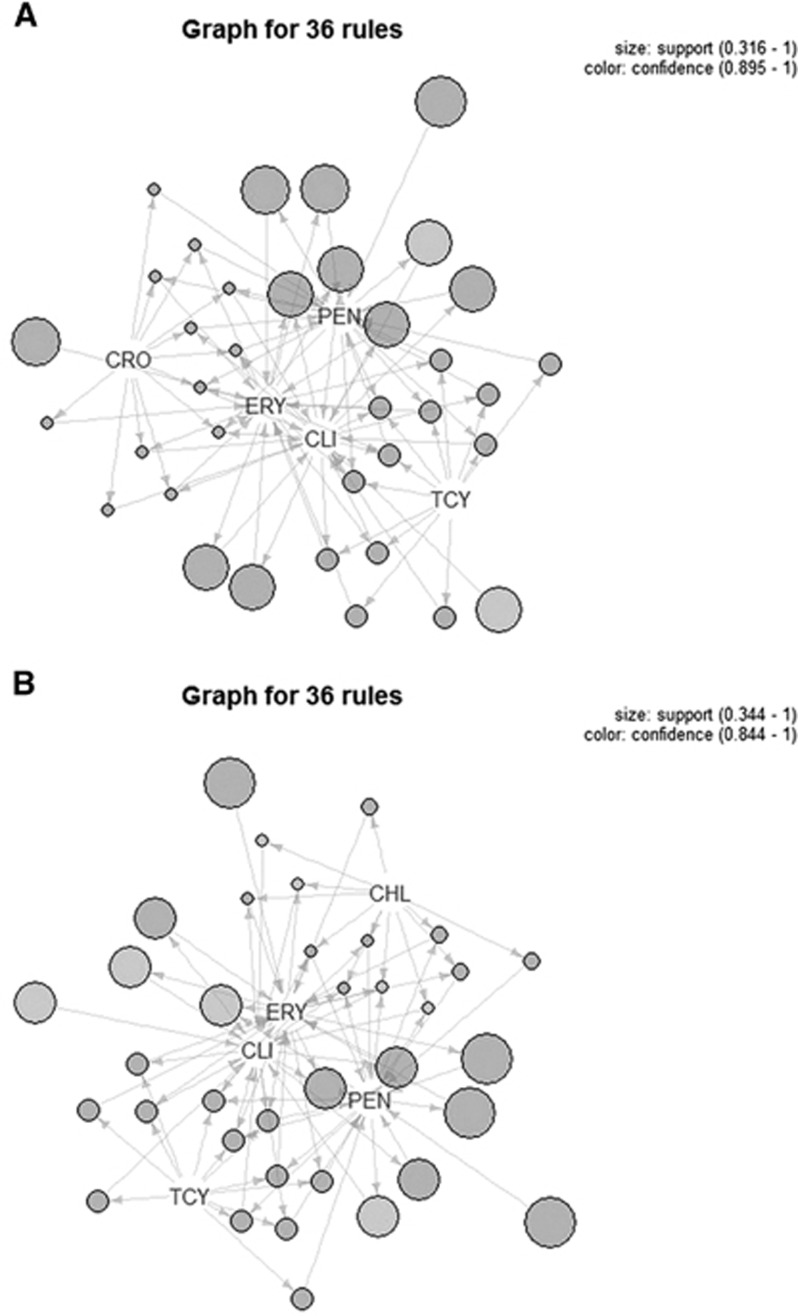
The association rule showed that there were 36 associations between the five antimicrobials in ST121 (**A**) and non-ST121 (**B**) CA-MRSA strains. Chloramphenicol, CHL; clindamycin, CLI; cefazolin, CZO; erythromycin, ERY; penicillin, PEN; tetracycline, TCY.

The 19 ST121 strains were isolated from patients with impetigo (13/19), cellulitis (1/19), secondary infection of eczema (3/19) and abscess (2/19). These 19 strains comprised nine *spa* types; however, only t437 was found in ST59 strains. There was no significant difference in antibiotic sensitivities between ST121 and non-ST121 MRSA isolates. Thirty-six separate associations between the five antimicrobials (erythromycin, penicillin, tetracycline, clindamycin and cefazolin/chloramphenicol) were found. Moreover, ST121 CA-MRSA strains tended to be more resistant to cefazolin, whereas non-ST121 CA-MRSA strains tended to be more resistant to chloramphenicol (Figure 1). Two mupirocin-resistant strains were observed among non-ST121 CA-MRSA strains.

## DISCUSSION

This study was conducted at 23 large, inner city, comprehensive medical teaching and research hospitals selected by the Chinese Ministry of Health to fill an important gap in the knowledge of community-associated *S. aureus* SSTIs in China. To our knowledge, this is the first large-scale comparison of CA-MRSA clinical characteristics between children and adults with *S. aureus* SSTIs in mainland China. We present three major findings: (i) the prevalence of CA-MRSA was low among children and adults with SSTIs; (ii) CA-MRSA strains in China have diverse genetic backgrounds and there is no trend of an outbreak thus far; and (iii) ST121 is currently the predominant clone.

The prevalence of CA-MRSA varies geographically, with a detection rate in patients with skin infections of 1–3% in France and up to 50% in the United States.^10^ The incidence of CA-MRSA SSTIs in China is unclear because of the lack of systematic epidemiological studies. A few small studies demonstrated that the incidence of CA-MRSA with SSTIs was 1.1–4% in Beijing and other regions of China.^11, 12^ In this study, we found the prevalence of CA-MRSA SSTIs to be surprisingly low, from 1.3% in North China to 6.1% in Southwest China. Furthermore, no MRSA infections were observed at two children's hospitals and four adult hospitals. A potential explanation for the differences in the prevalence of MRSA infections in distinct regions of China is most likely the vast size of the territories, along with disparities in economic development. Another explanation may be a data bias of fewer cases in these regions. The current absence of CA-MRSA outbreaks or reports on serious outcomes of CA-MRSA infections suggests that the low prevalence observed in this study may truly reflect the current situation in China.

Antibiogram testing is a crucial step in MRSA screening and selection of the appropriate antibiotic for proper treatment of skin infections. Our study showed that most patients had MSSA (1 964/2 716 patients). On comparing the antibiotic susceptibility patterns of bacterial isolates from children and adults, significant differences were found. Antibiotic susceptibility patterns therefore appear to depend mainly on bacterial strains, rather than type of skin infections. The majority of patients with SSTIs can be cured only using topical antibiotics; however, a small number of patients require systematic treatment. According to the results of antibiotic susceptibility tests in this study, penicillin and erythromycin are no longer appropriate agents, and cephalosporin may be the wiser choice. Although MRSA are generally resistant to all previously available beta-lactams, it was interesting that cephalosporin demonstrated potent activity against CA-MRSA. Indeed, traditional antistaphylococcal antibiotics (cephalosporin) have been associated with good clinical outcomes for CA-MRSA SSTIs. Recently, ceftaroline, which is a novel parenteral broad-spectrum cephalosporin, demonstrated bactericidal activity against Gram-positive organisms (including MRSA) and represented a bactericidal option for the treatment of MRSA infections, including those caused by isolates with reduced susceptibilities to vancomycin and daptomycin.^13^

Fusidic acid and mupirocin are effective topical antibacterial agents for the management of skin infections and *S. aureus* colonization in both patients and healthcare workers. In the past decade, there has been an increase in fusidic acid resistance in a number of countries. However, the fusidic acid resistance rates between MSSA and MRSA may differ. From 1998 to 2001, data from the United Kingdom showed that the resistance rates for fusidic acid in MSSA increased from 6.0% to 11.5%, whereas the resistance rates in MRSA were much lower and remained constant at ~2%.^14^ In this study, we also found it interesting that only MSSA was resistant to fusidic acid (1.4%). In 1999, fusidic acid was first introduced in China and has been available as a topical cream since 2003. However, resistance to fusidic acid in China was reported after 2007.^15, 16^ Therefore, selective pressure from antibiotic use likely led to increased resistance to fusidic acid. Similarly, initial reports on mupirocin-resistant *S. aureus* emerged shortly after the introduction of mupirocin into clinical practice.^17^ However, the prevalence of mupirocin resistance has increased in settings where this agent is used extensively. It has been reported that more than 50% of community-associated *S. aureus* strains displayed high-level resistance to mupirocin in Canada.^18^ In Beijing Children's Hospital, mupirocin ointment has been prescribed since 1993. However, only one strain was found to be resistant to mupirocin in 2005.^15^ In this study, the number of mupirocin-resistant *S. aureus* isolates clearly increased, but remained low compared with rates observed in other countries. Mupirocin therefore remains an effective therapy for the elimination of staphylococci in China.

It has been reported that five major CA-MRSA clones are disseminating worldwide. For example, the ST1 clone is observed in Asia, Europe, and the United States; the ST8 clone in Europe and the United States; the ST30 clone in Australia, Europe and South America; the ST59 clone in Asia and the United States; and the ST80 clone in Asia, Europe, and the Middle East.^19^ In 2007, Schefold *et al.*^20^ first reported a sepsis case of a 51-year-old male caused by *S. aureus* ST121. Thereafter, ST121 MSSA isolates were found to be distributed in 15 out of the 19 surveyed countries, namely Paraguay, New Caledonia, Togo, France, Czech Republic, Germany, Turkey, the United States, French West Indies, United Kingdom, Polynesia, Switzerland, Spain, Algeria and The Netherlands.^21^ A small study performed in mainland China demonstrated that ~8.3% (1/12) of SSTIs^22^ and 30.6% of asymptomatically colonized children in kindergartens^23^ were associated with ST121 isolates. These findings suggest that most ST121 strains are MSSA. The ST121 clone is rarely dominant in MRSA clinical infections. It has been reported that 11.8% of MRSA strains belonged to ST121 in Cambodia,^24^ 7.1% in Japan,^25^ 5.4% in Portugal^26^ and 0.3% in Spain.^27^ The important finding from the present study is that ST121 (35.3%), which had been previously documented in only two isolates (3.5%) among MRSA causing SSTIs in 2015,^28^ was more prevalent than ST59 (25.5%). Moreover, ST121 isolates have disseminated in seven hospitals located in five regions in mainland China. Interestingly, neither ST121 nor ST59 was observed in East China hospitals, an area of relatively rapid economic development. In addition, predominant *spa* type t437, which comprised 43.2% of all CA-MRSA, was also not observed in East China. There were also no significant differences in antibiotic sensitivities between MRSA and MSSA. MSSA has evolved as MRSA through the acquisition of SCC*mec,*^29, 30^ and therefore, some MSSA genotypes are the same as some popular CA-MRSA genotypes. Baines *et al.*^31^ found that ST5 MRSA clone has emerged from locally circulating ST5 MSSA strains in New Zealand. In addition, Stegger *et al.*^32^ determined that a single descendant of a PVL-positive methicillin-sensitive ancestor circulating in sub-Saharan Africa rose to become the dominant CA-MRSA clone in Europe, the Middle East and North Africa. The findings in this study indicate that the genetic background of CA-MRSA found in mainland China is complex. We will next probe more deeply into the genotyping of MSSA and expect to find clues of MRSA 'conversion' from MSSA in China.

The role of PVL in the pathogenesis of staphylococcal infections remains controversial. However, PVL has been closely associated with CA-MRSA infections, and there is a strong epidemiological association between carriage of *pvl* genes and successful CA-MRSA lineages. PVL-positive *S. aureus* strains are more frequently associated with cellulitis and abscesses than with impetigo.^33^ Although highly virulent CA-MRSA strains carrying *pvl* genes are known to prevail worldwide, the prevalence of PVL-positive MRSA strains from SSTIs in China was reported to range from 12.5% to 19.1%.^28, 34^ Compared with a previous study, the overall positivity rates of *pvl* genes in the present study was lower (9.8%), indicating a decreased prevalence of *pvl* genes among *S. aureus* SSTI isolates in China. Five patients with PVL-positive CA-MRSA infections were able to perform daily activities and had no identifiable risk factors. All five patients were empirically treated with cephalosporin and good outcomes were achieved.

In summary, this study provided information on the epidemiological and molecular characteristics of community-acquired *S. aureus* SSTIs among Chinese populations. The low prevalence of CA-MRSA is positive news. Interestingly, ST121 was the predominant clone among CA-MRSA strains. Moreover, there were no clear regional variations. To fully understand the epidemiology of *S. aureus* clone ST121, the continued systematic surveillance of both hospital- and community-associated isolates is required.

## Figures and Tables

**Table 1:** Demographic and clinical features of patients from children and adults with skin and soft-tissue infections

**Variables**	**Children**	**Adults**
*General information*		
Total patients (*n*)	2046	670
Male to female ratio	1.25:1	1.24:1
Age (mean±SD, years)	3.69±2.67	29.43±22.25
Range	1 d to 18 y	19–93 y
MSSA (*n*)	1705	241
MRSA (*n*)	44	7

*Types of skin and soft-tissue infection (n) (SA/MRSA)*		
* Diseases*		
Impetigo	1397/28	48/1
SSSS	82/2	0/0
Secondary infection of eczema	74/2	58/1
Abscess	28/5	13/3
Secondary infection of urticaria-like papules	27/0	0/0
Folliculitis	21/1	27/0
Furuncle	10/1	14/1
Cellulitis	9/2	2/0
Paronychia	4/1	0/0
Trauma	4/0	8/1
Omphalitis	2/1	0/0
Necrotizing fasciitis	1/0	0/0
Other secondary infections	46/1	71/0
**Region**	**Hospital**	**(*n*) (patient/SA/MRSA)**
*Source and distribution of patients*		
Northeast	HBCH	187/142/6
	CCCH	23/10/2
	DLCH	9/8/0
North China	PPH	35/8/0
	PTH	30/11/0
	PFH	71/28/2
	BJCH	826/787/9
	SXCH	90/69/1
	TJCH	5/5/0
East China	HSH	18/6/0
	XZCH	204/163/2
	FDPH	35/26/1
Northwest	XJH	94/34/1
	UFH	89/52/1
	XJFH	50/33/1
Southwest	WCH	90/17/1
	SWH	94/46/0
	CQCH	116/102/9
South Central China	WHH	66/11/1
	XYH	83/28/1
	ZZCH	196/153/3
	HNCH	131/82/2
	GZCC	174/125/8

Abbreviations: methicillin-resistant *Staphylococcus aureus*, MRSA; methicillin-sensitive *S. aureus*, MSSA; *Staphylococcus aureus*, SA.

Three letter abbreviations for adult's hospital and four letter abbreviations for children's hospital: Beijing Children's Hospital, BJCH; Children's Hospital of Changchun, CCCH; Children's Hospital of Chongqing Medical University, CQCH; Dalian Children's Hospital of Dalian Medical University, DLCH; The Paediatric Hospital of Fudan University, FDPH; Harbin Children's Hospital, HBCH; Hunan Children's Hospital, HNCH; Huashan Hospital of Fudan University, HSH; Guangzhou Women and Children's Medical Center, GZCC; Peking University First Hospital, PFH; Peking University; People's Hospital, PPH; Peking University Third Hospital, PTH; The First People's Hospital of Urumqi, UFH; Southwest Hospital, SWH; Children's Hospital of Shanxi, SXCH; Tianjin Children's Hospital, TJCH; West China Hospital, WCH; Wuhan No.1 Hospital, WHH; The First Affiliated Hospital of Xinjiang Medical University, XJFH; Xijing Hospital, XJH; The Second Xiangya Hospital of Central South University, XYH; Xuzhou Children's Hospital, XZCH; Zhengzhou Children's Hospital, ZZCH.

**Table 2:** Results of the susceptibility testing of the strains

**Agents**	**Children (*n*=1705)**	**Adults (*n*=241)**	***P***	**MRSA (*n*=51)**	**MSSA (*n*=1895)**	***P***
	**%*R***	**%*R***		**%*R***	**%*R***	
Penicillin G	96.8	87.9	*P*≤0.05	100	96.6	0.412
Erythromycin	96.8	86.6	*P*≤0.05	98	97.3	1
Clindamycin	89.3	70.7	*P*≤0.05	86.3	89.1	0.528
Tetracycline	38.8	31.8	0.041	49	42	0.317
Chloramphenicol	15.1	24.8	*P*≤0.05	31.4	14.4	*P*≤0.05
Gentamicin	9.6	29.3	*P*≤0.05	5.9	9.9	0.339
Ciprofloxacin	6.2	20.4	*P*≤0.05	13.7	6.3	*P*≤0.05
Trimethoprim/sulfamethoxazole	3.6	7.6	0.004	7.8	4.5	0.291
Oxacillin	2.6	1.9	0.639	100	0	/
Fusidic acid	1.8	7	*P*≤0.05	0	1.4	1
Rifampin	1.7	2.7	0.194	3.9	2.4	0.351
Cefazolin	1.4	6.4	*P*≤0.05	3.9	1.6	0.204
Mupirocin	1.3	5.8	*P*≤0.05	3.9	0.7	0.057
Ceftriaxone	1.1	4.5	*P*≤0.05	23.5	0.9	*P*≤0.05
Cefixime	0.5	5.7	*P*≤0.05	19.6	2.5	*P*≤0.05
Vancomycin	0	0	/	0	0	/

Abbreviations: methicillin-resistant *Staphylococcus aureus*, MRSA; methicillin-sensitive *S. aureus*, MSSA.

**Table 3:** Clinical and molecular features of 51 cases with CA-MRSA infections

**Number**	**Case**	**Hospital**	**Infection**	**MLST**	**SPA**	**SCC*mec***	**PVL**
1	XZ40A	XZCH	Impetigo	st1	t127	V	−
2	C490	BJCH	Impetigo	st121	t1425	IV	−
3	C668	BJCH	Impetigo	st121	t2086	IV	−
4	C958	BJCH	Impetigo	st121	t2086	V	−
5	C732	BJCH	Impetigo	st121	t437	V	−
6	C1243	BJCH	Impetigo	st338	t437	V	−
7	C871	BJCH	Impetigo	st448	t1425	IV	−
8	C867	BJCH	Paronychia	st59	t437	IV	−
9	C1252	BJCH	Impetigo	st59	t437	V	−
10	C152	BJCH	Impetigo	st59	t437	IV	−
11	CC18	CCCH	Impetigo	st121	t2019	IV	−
12	CC16	CCCH	Impetigo	st573	t1839	V	−
13	CCH40	CQCH	Impetigo	st121	t1425	V	−
14	CCH42	CQCH	Impetigo	st211	t437	V	−
15	CCH115	CQCH	Abscess	st45	t2086	V	−
16	CCH184	CQCH	Abscess	st59	t437	IV	−
17	CCH229	CQCH	Impetigo	st59	t437	V	−
18	CCH118	CQCH	Abscess	st121	t2086	IV	+
19	CCH302	CQCH	Cellulitis	st121	t437	IV	+
20	CCH193	CQCH	Abscess	st59	t437	V	+
21	CCH117	CQCH	Abscess	st121	t437	IV	
22	EK16	FDPH	Impetigo	st8	t008	V	−
23	GCH43	GZCC	Impetigo	st121	t1425	IV	−
24	GCH36	GZCC	Impetigo	st121	t2086	IV	−
25	GCH291	GZCC	Impetigo	st121	t269	IV	−
26	GCH92	GZCC	Impetigo	st121	t375	V	−
27	GCH268	GZCC	Impetigo	st121	t8660	V	−
28	GCH52	GZCC	Secondary infection of herpes simplex	st338	t437	V	−
29	GCH101	GZCC	SSSS	st448	t437	IV	−
30	GCH35	GZCC	Furuncle	st338	t437	V	+
31	HCH37C	HBCH	Impetigo	st121	t127	IV	−
32	HCH249	HBCH	Secondary infection of eczema	st121	t2086	V	−
33	HCH233	HBCH	Secondary infection of eczema	st121	t437	V	−
34	HCH68C	HBCH	Impetigo	st211	t1425	V	−
35	HCH23	HBCH	Impetigo	st59	t437	IV	−
36	HCH65C	HBCH	Impetigo	st59	t437	V	−
37	HN104	HNCH	Omphalitis	st19	t30	V	−
38	HN133	HNCH	Cellulitis	st20	t2919	V	
39	BHC108	PFH	Impetigo	st88	t7637	IV	−
40	BHC123	PFH	Abscess	st59	t437	IV	+
41	SX75	SXCH	Impetigo	st121	t2086	IV	−
42	XJC41	UFH	Impetigo	st59	t437	IV	−
43	CDL13	WCH	Trauma	st1	t127	IV	−
44	WH54	WHH	Abscess	st59	t437	IV	−
45	XJH31	XJFH	Furuncle	st59	t437	IV	−
46	XJ74	XJH	Secondary infection of eczema	st121	t114	IV	−
47	XYH34	XYH	Abscess	st59	t437	V	−
48	XZ107	XZCH	Folliculitis	st88	t325	IV	−
49	ZCH13C	ZZCH	Impetigo	st1	t127	V	−
50	ZCH03	ZZCH	Impetigo	st1	t1784	IV	−
51	ZCH18A	ZZCH	SSSS	st93	t202	V	−

Abbreviations: community-associated methicillin-resistant *Staphylococcus aureus*, CA-MRSA; multilocus sequence typing, MLST; panton-valentine leukocidin, PVL; Staphylococcal cassette chromosome *mec*, SCC*mec*; staphylococcal protein A, SPA.
